# Trap-Neuter-Return Activities in Urban Stray Cat Colonies in Australia

**DOI:** 10.3390/ani7060046

**Published:** 2017-06-02

**Authors:** Kuan Tan, Jacquie Rand, John Morton

**Affiliations:** 1 School of Veterinary Science, The University of Queensland, Gatton, Queensland 4343, Australia; j.rand@uq.edu.au (J.R.); john.morton@optusnet.com.au (J.M.); 2 Australian Pet Welfare Foundation, Kenmore, Queensland 4069, Australia; jacquie@petwelfare.org.au; 3 Jemora Pty Ltd., Geelong, Victoria 3220, Australia

**Keywords:** trap, neuter, return, urban stray, cats, desex, wildlife, euthanasia, control

## Abstract

**Simple Summary:**

Urban stray cats in Australia are poorly regarded because of wildlife predation and nuisance behaviors. However, current methods of population control via low level culling are ineffective. Effective control requires culling 30% to 50% of the population every six months, which is prohibitive for municipalities. Overseas, trap, neuter and return is frequently used to control urban cat numbers, and reduce nuisance behaviors, but is considered illegal in many Australian jurisdictions. An anonymous questionnaire was used to gather data on trap, neuter and return of urban stray cats in Australia. Respondents were mostly middle-aged women, and more participated as individuals than with organizations. Colony size decreased from a median of 11.5 cats to 6.5 cats over 2.2 years, through adoptions and desexing a median of 69% of the colony. Cats were fed once or twice daily, and provided with prophylactic health care. Programs were largely funded by private sources, with some funding by animal welfare organizations. We conclude that trap, neuter and return associated with high desexing rates in colonies, and adoption of kittens and friendly adults substantially reduces colony size, and improves the welfare of cats and kittens. This model is cost-effective for municipalities, and should be legalized in Australia.

**Abstract:**

Trap, neuter and return (TNR) describes a non-lethal approach to the control of urban stray cat populations. Currently, in Australia, lethal control is common, with over 85% of cats entering some municipal pounds euthanized. No research has been published describing TNR activities in Australia. Adults involved with TNR in Australia were invited to participate. Data from 53 respondents were collected via an anonymous online questionnaire. Most respondents were females 36 to 65 years of age, and slightly more participated in TNR as individuals than as part of an organization. Respondents generally self-funded at least some of their TNR activities. The median number of colonies per respondent was 1.5 (range 1 to over 100). Median colony size declined from 11.5 to 6.5 cats under TNR over a median of 2.2 years, and the median percent reduction was 31%; this was achieved by rehoming cats and kittens and reducing reproduction. A median of 69% of cats in each colony were desexed at the time of reporting. Most respondents fed cats once or twice daily, and at least 28% of respondents microchipped cats. Prophylactic healthcare was provided to adult cats and kittens, commonly for intestinal parasites (at least 49%), and fleas (at least 46%); vaccinations were less common. Time-consuming activities for respondents were feeding (median 4 h/week) and locating resources (median 1.1 h/week). These findings indicate that TNR, when involving high desexing rates within colonies, adoption of kittens and friendly adults, and ongoing oversight by volunteer caretakers, can reduce cat numbers over time, improve health and welfare of cats and kittens, and is largely funded by private individuals and organizations.

## 1. Introduction

Urban stray cats are often poorly regarded in the community because of wildlife predation, nuisance behavior, zoonotic concerns and fear of transmission of infectious diseases to pet cats [1,2]. Apart from wildlife predation and nuisance behavior, other reasons are not supported by the scientific literature [1,3,4]. Management strategies often used for feral cats include exclusion fencing, shooting, release of infectious disease, and toxins; these methods are not suitable for use in urban areas [5].

Approximately 3% to 5% of the Australian urban stray cat population is euthanized annually, based on estimates of cat admissions to shelters and municipal pounds annually (164,000 cats) and the percent euthanized (56%) [2]. This cull rate is based on assumptions that approximately half the 164,000 cat admissions are to municipal pounds, of which 85% are stray, half are to welfare agencies of which 60% are stray, and overall 56% of admitted cats are euthanized [2,6]. Approximately 90% of Australia’s 23 million residents live in urban areas where there are likely 60 to 100 stray cats per 1000 residents [7,8,9,10,11] which equates to between 1.2 and 2 million urban stray cats. Although there are no national data available, NSW municipal pound data show a steady slow annual increase in intake of cats and numbers killed [2,12], suggesting that the current low level killing of excess stray cats is ineffective in decreasing cat numbers. Culling increases kitten survival rates [13], and in some cases, low level culling increases cat numbers by a factor of 2–3 times due to immigration into the area and increased survival of juveniles [6]. To achieve attrition of open cat populations (i.e., populations where immigration and emigration can occur) requires an estimated 30% to 50% of the population to be trapped and culled every six months for at least a decade [13]. This magnitude of trapping for culling would require substantial labor and capital. In one study, it required a mean of nine trap-nights per cat to trap 90% of stray cats in a colony [14,15] with traps checked once or twice daily [15]. Culling of a magnitude sufficient to substantially reduce urban cat populations would also have significant social and human welfare implications. Approximately 50% of the workers involved with euthanasia of animals develop post-traumatic stress which is associated with depression, substance abuse, hypertension, sleeplessness and suicide [16,17,18,19]. 

Given the disadvantages of culling, alternative strategies need be considered, particularly when there is semi-ownership of cats. Semi-ownership occurs where some care, usually food, is provided by people who do not perceive they own the cat, and is common in Australia. In an Australian internet study, 9% of respondents daily fed a cat they did not perceive they owned, and a phone survey of Victorian residents found that 22% of households provided some care for a cat they said was not theirs [20,21,22]. In both studies, most of these semi-owned cats were not desexed. Semi-owned cats are a substantial contributor to the approximately 80% to 90% of stray cats entering shelters or pounds that have some socialization to humans, and therefore not considered feral [21,23,24].

Desexing is an important aspect of reducing shelter intake, and when stray cats are trapped, desexed and returned to their original location, it is referred to as trap, neuter and return (TNR.) TNR is used in many countries overseas including Singapore, Canada, Italy and the United States [15,25,26,27]. In North America, intake of stray cats into municipal pounds and shelters and their euthanasia are markedly decreased where targeted TNR is employed [9]. 

Currently in Australia, rescue groups and individuals unofficially trap, neuter and return cats, often also providing ongoing care of the colony through feeding and monitoring. Reductions in colony size after introduction of TNR have been reported in colonies in USA [15,28,29,30]. However, there is no published evidence for the efficacy of TNR in reducing colony size in Australia. Legally, the return of cats could be considered an offence in Australia under legislation relating to abandonment of cats, and cats as pest species. Specifically, the Prevention of Cruelty to Animals Act 1979 (Section 11, and the National Parks and Wildlife Act 1974 (Sections 109 and 133(4)) amongst others relate to TNR. The aims of this study were to describe TNR activities in Australia, and to estimate their effect on colony size.

## 2. Materials and Methods

### 2.1. Study Design Overview

A cross-sectional study was conducted with adults involved in TNR in urban areas in Australia enrolled using convenience sampling, including snowball sampling. Each participant could report results for one or multiple colonies of cats with which they were involved. Data were collected using an online questionnaire created using online survey software (Survey Monkey^®^, Survey Monkey, San Mateo, CA, USA) and a downloadable Microsoft Word version (Microsoft Corporation, Redmond, WA, USA) was provided for anonymous postal responses for respondents concerned about traceability of IP addresses. The survey contained open and closed questions on information of TNR sites and colonies, demographics of the respondent, their motivations for involvement, TNR operations including feeding, trapping, desexing, identification, healthcare, rehoming, funding and costs. The survey was approved by Bellberry Human Ethics Committee EC00450. 

### 2.2. Data Collection and Eligibility Criteria

A link to the online questionnaire and a downloadable document were hosted on the website of an Australian not-for-profit organization involved with rehoming cats and dogs (Maggie’s Rescue, New South Wales), and disseminated via email to their contact list. Potential respondents were requested to complete the questionnaire if they were involved with TNR, and to forward it on to others they knew who were involved in TNR, utilizing a “snowballing” effect. In the questionnaire introductory text, adults were invited to participate “in this survey about trap, neuter and return (TNR) activities to manage cats living in the community”. These cats were described as “community cats”. The scope was described as including “colonies where cats are managed in some way after desexing and returning, and those that are not managed afterwards. It does not include colonies where desexing does not occur”. People were asked to complete the questionnaire only if the TNR was conducted in urban areas, and not in bushland, National or State parks or reserves. Respondents were asked whether they were aged 18 years and over or not, and those selecting no were prevented from completing the questionnaire. To maximize response rate, a modified form of Dillman’s Tailored Method was utilized where three email reminders were sent at approximately one-week intervals. No inducements were offered to participants completing the questionnaire. 

As TNR activities might be interpreted as illegal in some situations with respect to legislation associated with pest species and abandonment, participants were advised of the potential that TNR could be considered an offence in some jurisdictions and advised if they had concerns not to complete the online survey study but to download a Word version and submit anonymously by post to Maggie’s Rescue.

### 2.3. Statistical Analyses

Statistical analyses were performed using Stata (version 14, StataCorp, College Station, TX, USA). The percent reduction in each colony’s size was calculated as (initial − current number of cats)/initial number of cats). Changes in the number of cats in the colony from the commencement of TNR to the time of reporting were compared using Wilcoxon’s matched pairs signed rank tests. Distributions of values for colonies were compared between colonies where TNR had been used for ≤2 years or >2 years, and between colonies under individual- and organization-operated program, using Somers’ D without transformation; 95% confidence intervals and *p*-values were calculated based on jackknife standard errors adjusted for clustering of colony within respondent and the z-distribution. Correlations between other colony-level variables were assessed using Spearman’s rank correlation coefficients not adjusted for clustering of colony within respondent. Fisher’s transformation was used to calculate 95% confidence intervals for these correlation coefficients. Two-sided *p*-values were used.

## 3. Results

### 3.1. Number of Respondents and Colonies, Respondent Demographics and Colony Locations

A total of 53 responses were received between 22 September and 16 December 2016. One of these respondents completed the agreement sections but provided no other data and so was excluded. The remaining 52 respondents provided information about the number of colonies they managed and/or colony locations. Of these, 13 respondents provided no other information so, for other analyses, the study population consisted of 39 respondents. Not all of these 39 respondents answered all questions; numbers of responses are shown for all results, and percentages are calculated of those that responded. A small number of responses were disregarded where multiple discordant values were provided for the same question.

**Figure 1:**
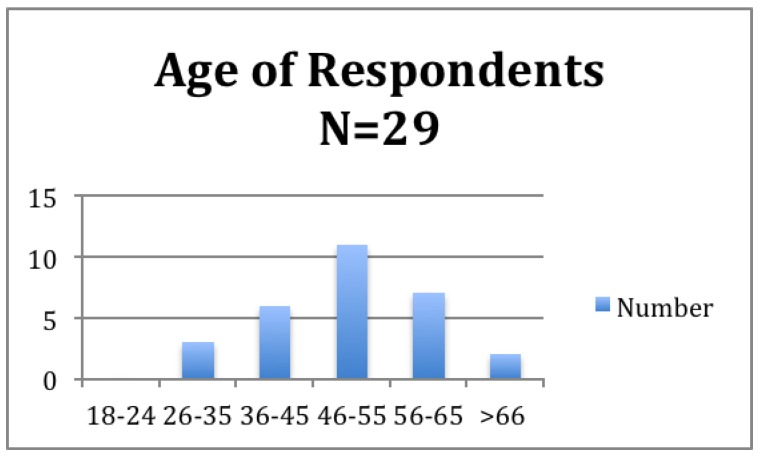
Age distribution of respondents.

Most respondents were female (90% or 27/30), and the peak age category was 46–55 years (38% or 11/29; Figure 1). More respondents were involved in TNR as individuals (58% or 29/50) rather than as part of an organization (42% or 21/50). 

Each respondent was involved with a median of 1.5 colonies (range 1 to over 100; *n* = 48 respondents). Each of the respondents in individual-operated programs were involved with between 1 and 10 colonies (median 1; *n* = 28 respondents), while those in organization-operated programs were involved with between 1 and over 100 colonies each (median 3.5 colonies; *n* = 20 respondents). Most respondents were involved with colonies in New South Wales (53% or 26/49 respondents) and Victoria (22% or 11/49 respondents. Distributions of colony locations were similar for individual- and organization-operated colonies (Table 1). In total, 64 postcode locations of colonies were nominated by 38 respondents. Nearly all (97% or 62/64) were in major urban locations based on the classification of the Department of infrastructure and Regional Development [31]. Other locations were bounded locality (town) (*n* = 1) and other urban (regional city) (*n* = 1).

In total, 98 colony location descriptions were reported by 50 respondents. The most common location descriptions were private residential homes (26%), industrial areas or factory complexes (20%) and alleyways or streets (13%) (Table 2). Distribution of locations was similar for individual- and organization-operated programs.

### 3.2. Number of Colonies, Colony Characteristics and Changes over Time

Thirty-two respondents provided colony population size and composition data for 55 colonies. Four of these colonies had no desexed cats at the time the respondent completed the questionnaire. These were deemed to not be under TNR management and were excluded from the study leaving 51 colonies. Of these, for 44 colonies from 28 respondents, unambiguous data (i.e., only one value rather than multiple discordant responses) were provided for initial and current numbers of cats; each of these 28 respondents provided data for between one and four colonies (median 1). Median initial colony size (i.e., at the time TNR was commenced) for these 44 colonies was 11.5 cats (range 3 to >50). At the times the respondents completed the questionnaire, TNR had been used for a median of 2.2 years (range 0.1 to 12 years; 33% or 13/40 were <1 year; n for duration of TNR = 40 colonies), the median number of cats was 6.5 (range 1 to >50); the distribution of colony sizes differed significantly from initially (*p* < 0.001).

Absolute and percent reductions could not be calculated for two colonies as they were recorded as having “>50” cats both initially and currently. For the remaining 42 colonies, the number of cats had declined in 32 colonies, not changed in seven colonies and increased in five colonies. The median percent reduction was 31% (range 77% increase to >90% reduction *n* = 42 colonies). After pooling the 37 of these colonies where specific initial and current numbers of cats and time were recorded, total initial and current numbers of cats were 515 and 344, respectively, a reduction of 33%. Median time TNR had been used for these 37 colonies was 2.4 years.

The time TNR had been used was recorded for 38 of the 42 colonies where percent reduction could be calculated. The percent reduction was positively correlated with time TNR had been used (Spearman’s r 0.47; 95% CI 0.18 to 0.69; *p* = 0.002). For the 18 colonies where TNR had been used for two years or less, the median percent reduction was 20% (range 76% increase to 90% reduction, median time 0.5 years; range 0.1 to 2.0 years). In contrast, for the 20 colonies where TNR had been used for more than 2 years, the median percent reduction was 49% (range 67% increase to >90% reduction; *p* = 0.006, median time 6.0 years; range 2.4 to 12.0 years).

Twenty-two of these 44 colonies were under individual-operated programs. Their median initial colony size was 11 cats while at the time of reporting, TNR had been used for a median of four years, and the median number of cats was six (P for comparison of colony sizes <0.001). The number of cats had declined in 17 colonies, was not changed in four colonies and had increased in one colony. The median percentage decline was 40% (*n* = 21 colonies). The remaining 22 of these 44 colonies were under organization-operated programs. Their median initial colony size was 13.5 cats, and at the time of reporting, TNR had been used for a median of 0.6 years and the median number of cats was 7.5 (P for comparison of colony sizes 0.004). The number of cats had declined in 15 colonies, not changed in three colonies and increased in four colonies. The median percent reduction was 24% (*n* = 21 colonies).

For 21 colonies reported by 14 respondents, all reported numbers were unambiguous and none were discordant (i.e., current numbers of adult cats and kittens summed to the reported current numbers of cats, as did numbers of males, females and sex unknown, and the current number desexed was not greater than the current numbers of cats). Results for these 21 colonies are reported in Table 3. 

At the time of reporting, colonies with unambiguous data (*n* = 21) consisted predominantly of adult cats (median 100%). Proportions of cats that were desexed varied widely between colonies (median 69%), as did proportions of cats that were male (median 50%). Numbers of cats in colonies at the commencement of TNR did not differ significantly between colonies with individual- versus organization-operated programs, but more cats were removed from colonies with individual-operated programs, and a lower proportion of cats were male at the time of reporting (Table 3). The median percent desexed for individual-operated programs was 100% compared to 67% for organization-operated programs (*p* = 0.192).

The difference in number of cats that had been removed may have been, in part, because in the eight colonies with individual-operated programs, TNR had been used for longer than in the 11 colonies with organization-operated programs, where time was recorded (median 3.2 years versus 1.0 year). The number of cats removed was positively correlated with time TNR had been used (Spearman’s r 0.42; 95% CI −0.04 to 0.74; *p* = 0.072). The estimated correlation coefficient for the correlation between the number of cats removed, expressed as a ratio of the number of cats in colony at commencement of TNR, and time was very imprecise and inconclusive (Spearman’s r 0.25; 95% CI −0.23 to 0.63; *p* = 0.310). There was no evidence of a close association between the proportion of cats that were male and time (Spearman’s r −0.02; 95% CI −0.47 to 0.44; *p* = 0.923). 

Across these 21 colonies, the median percent reduction was 40% (range 77% increase to 83% reduction). The percent reduction was positively correlated with the number of cats removed, expressed as a ratio of the number of cats in colony at commencement of TNR (Spearman’s r 0.62; 95% CI 0.26 to 0.83; *p* = 0.003). The correlation coefficient for the association between percent reduction and time since TNR commenced was 0.43 (95% CI −0.03 to 0.74; *p* = 0.065), and between percent reduction and current percent desexed was 0.29 (95% CI −0.16 to 0.64; *p* = 0.199). The number of cats had declined in 16 colonies, not changed in 1 colony and increased in 4 colonies. In the 16 colonies where numbers had declined, medians of time since TNR commenced, number of cats removed, expressed as a ratio of the number of cats in colony at commencement of TNR, and current percent desexed were 3.2 years, 0.56 removed ratio and 75% currently desexed, compared with 0.5 years, 0.00 removed ratio and 65% desexed for the five colonies where numbers had not declined.

### 3.3. Reasons Respondents Became Involved in TNR

The main reasons for becoming involved with TNR were to stop cats breeding (77%), to make life better for stray cats (67%) and because the cats were at risk of being trapped and killed by authorities (60%) (Table 4). Only 20% said it was because the stray cats were a nuisance. Reasons for involvement in TNR were similar for respondents from individual- and organization-operated programs (Table 3).

Additional comments from respondents demonstrated that their personal concerns for cat welfare shaped their individual responses to cats they believed to be disadvantaged. For example “the colony in my street were breeding, and there were so many kittens and many died. It was a mess. I had to learn TNR. Now the colony is small and many have been rehomed” and “breeding was rampant and it wasn’t possible to bring all cats into foster care for rehoming”. 

### 3.4. Roles Undertaken at the Colonies by Respondents and Time Involvement

For those respondents who conducted the activity, the most time-consuming activities for them at the time they completed the questionnaire were feeding cats (median 4 h/week), organizing feeding rosters (median 1.5 h/week), and locating resources (median 1.1 h/week) (Table 5). For many activities, the range of hours between respondents was very large. Medians for finding homes for cats and/or kittens, and for trapping, were much higher in individual-operated programs than in organization-operated programs. 

For all those who provided care pooled, for the largest colony, the most time-consuming activities were feeding cats (median 6.5 h/week), locating resources (median 1.8 h/week), and developing or maintaining community relationships (median 1.5 h/week). Again the range between colonies was large for many activities (Table 3). 

The activities that involved the most people were feeding cats (3 people in any one week), organizing feeding roster (1 person in any one week) and locating resources (0.6 people in any one week). There was large variability between colonies for some activities; for example, returning cats to their original location over six months involved between 1 and 100 people in organization-operated programs. 

### 3.5. Feeding and Shelter Provision

Of the 39 respondents who answered questions in addition to those about the number of colonies they managed and/or colony locations, 25 (64% or about two-thirds) answered at least one question indicating they fed cats at a TNR colony they reported on. In individual-operated programs, similar proportions of respondents fed once and twice daily, but most respondents in organization-operated programs fed once daily (Table 4). There was no predominant feeding time. About two-thirds of respondents used specialized feeding stations that hid food, and most respondents placed the food in a container on the ground or uncontained on the ground. Most respondents left food to be eaten at any time. More respondents involved in individual-operated programs provided shelter, and this included cardboard boxes and dog kennels, while only a minority of respondents in organization-operated programs provided shelter (Table 6).

### 3.6. Trapping

Of the 25 respondents who answered at least one question indicating they trapped cats at a TNR colony they reported on, 16 were involved with individual- and 9 with organization-operated programs. Thus, for all 39 respondents, the minimum percentage of people involved in trapping was 25/39 or 64%. Commercial traps such as ACES cat trap (discontinued Chinese manufacturer), Tomahawk (Tomahawk Live Trap, Hazelhurst, WI, USA), collapsible drop trap, and foot pedal traps were used by most respondents that trapped in individual- (11/16, 69%) and organization-operated (7/9, 78%) programs. Less commonly (28%), a mixture of home-made and commercial traps was used by respondents who trapped (7/25).

The majority of 21 respondents who reported on preparation of cats for trapping, conditioned cats to a regular feeding schedule (19/21, 90%). Respondents varied widely in the typical number of nights it took to trap a cat. If cats were not conditioned to a feeding schedule, while 50% (7/14) reported two nights or fewer, others reported 2–3 or seven nights. In contrast, if cats were first put on a feeding schedule, most respondents (80%, 16/20) reported that the normal number of nights it took to trap a cat was two nights or fewer. Someone sat nearby until the cat was trapped for 85% (11/13) of respondents that trapped with individual-operated programs, and for all nine respondents that trapped with organization-operated programs. Otherwise, in individual-operated programs, traps were checked twice (1 respondent) or three times (1 respondent) within a 24-hour period.

### 3.7. Desexing

Of the 39 respondents, 25 (64%) answered at least one question indicating they had knowledge of desexing of cats at a TNR colony with which they were involved. Of these respondents, most (79% or 11/14) who were involved with individual-operated programs and all nine respondents involved with organization-operated programs said an attempt was made to desex most of the colony over a short period. However, only 50% (10/20) of respondents indicated that all or nearly all the cats from the colony were desexed by six months after commencement of TNR; 15% of the respondents (3/20) indicated this took one month, 20% (4/20) took 2–3 months and 15% (3/20) took 5–6 months. In the period until all or nearly all were desexed, 60% (12/20) of respondents indicated that 80 to 100% were trapped in this period, whereas others indicated it was only three-quarters of the colony (15% or 3 respondents), half of the colony (10% or 2 respondents), quarter of the colony (10% or 2 respondents) or only a few cats (1 respondent).

Of respondents (*n* = 24), all indicated that private veterinary clinics performed the desexing, and two of these respondents also used not-for-profit pet welfare organizations for desexing. After including respondents who indicated desexing cost was $0, and after excluding implausibly high reported desexing costs (≥$1500 per cat), the median cost of desexing males was $60 (range $0 to $300; *n* = 20 respondents), for females, $100 ($0 to $300; *n* = 19) and for pregnant females $115 ($0 to $230; *n* = 13). After desexing, queens older than six months of age were returned to their original location after a median of four days (range same day to >7 days; *n* = 24 respondents). In contrast, males older than six months of age were returned after a median of one day (range same day to >7 days; *n* = 24 respondents). Just under half of these 25 respondents reported that cats were microchipped when they were desexed (44% or 11/25), equating to at least 28% of the 39 respondents, and respondent details were recorded on the microchip database by nine of these 11 (82%). Rescue group and/or organization details were concurrently recorded on the database by 4/11 (36%) respondents. Only 33% (9/27) of respondents indicated that cats were ear tipped when desexed, but 50% (7/14) of respondents who did not microchip instead ear-tipped, and four of the remaining seven reported using tattooing or photographs.

### 3.8. Healthcare

Of the 39 respondents, 26 respondents reported that they used at least one of the eight nominated routine health care strategies for kittens or cats, or provided health care when required at a colony with which they were involved. The majority of these 26 respondents indicated they routinely treated for fleas and intestinal parasites in adults and kittens (Table 7). A majority of respondents said they routinely vaccinated kittens with F3 and/or F4 (62%) but less did this routinely for adult cats (38%). One third tested for Feline Immunodeficiency Virus (FIV) in kittens but only a minority tested adult cats. 

Of the five respondents not using routine treatment for fleas in either adult cats or kittens, one treated as necessary, and of the four respondents not using routine treatment for intestinal parasites in either adult cats or kittens, two treated as required. Of the 26 respondents, the majority reported they provided health care when necessary for abscesses (65% or 17), minor wounds (62% or 16) and cat flu (58% or 15), but only 38% (10) treated ringworm. Most respondents (88% or 22/25) indicated that medical records were kept. 

### 3.9. Rehoming and Returning

Most respondents (81% or 21/26) reported it was their or their organization’s practice to remove adoptable adult cats for adoption, and if necessary, socialization. For the remaining 19% (5/26) of respondents to this question, the policy was to return adoptable adult cats to their original location. Most respondents said it was their or their organization’s practice to remove kittens up to eight weeks and 8–12 weeks of age (92% or 24/26 for each) for adoption and socialization, while the remaining respondents desexed and returned kittens to their original location. For kittens older than 12 weeks of age, 79% (19/26) of respondents said their policy was for removal and adoption, and if necessary, socialization.

In total, 68% (19/28) indicated there were attempts to rehome adoptable adult cats from the colony in the previous 12 months. For kittens, for seven of 27 respondents, their colony did not have kittens born in the last 12 months. Of the remaining 20 respondents, 19 (95%) attempted to rehome kittens. Where there were such attempts, a median of 3.5 adult cats (range 1 to 100; *n* = 18) and 8.0 kittens (range 1 to approximately 60; *n* = 17) were fostered or adopted.

### 3.10. Funding

The majority of respondents involved with individual-operated programs contributed either by cash and/or in-kind contributions for each of transport, desexing, traps, bedding and food (Table 6). However, private sources (other than the respondent) and/or organizations also assisted with cash and/or in-kind support for desexing (44% of respondents), and traps and food (25% for each). For respondents involved in organization-operated programs, less respondents self-funded transport, desexing, traps, and food, and for most or all, private sources and/or organizations assisted for all activities except transport. The majority of respondents self-funded transport (67% of respondents) and bedding (86% of respondents) (Table 8). Respondents commented that commercial traps were supplied by some local animal welfare organizations.

## 4. Discussion

This cross-sectional study represents pioneering research into TNR activities in urban Australia and adds to the small body of international peer-reviewed literature on this issue. Our study has several points of difference to previously published international research. Importantly, in contrast to studies conducted overseas, our study collected data on TNR activities undertaken by many individual members of the public, and just over half participated as individuals not associated with any organization.

### 4.1. Respondent Demographics and Colony Location

Nearly all participants were female (90%) and most were aged 36 to 65 years old. Survey studies where participants are self-selected typically have disproportionately higher female participation [32]. Propagation via “snowballing” and use of social media may have contributed to an overrepresentation of females in the study population relative to all those involved in TNR in Australia. However, cat feeders are typically female [33], and therefore females may not be over-represented in the study population. In a study of 3107 randomly selected respondents weighted to represent the American population, women had more humanistic and moralistic attitudes towards animals than men [34,35], and women show greater attachment to companion animals than males [35].

Nearly all colonies were in major urban locations, and most capital cities in Australia were represented by respondents engaged in TNR, with private residences, industrial areas and alleyways or streets the most common colony sites. Similarly, a New Zealand-wide study found 75% of urban stray cat colonies were located within residential zones, 10% within commercial zones and 12% shared between a hospital, schools and restaurants, and the remainder at countryside and beach locations [36]. In Tel Aviv, Israel, higher densities of adult stray cats were found in neighborhoods with a mix of residential and commercial areas compared with solely residential neighborhoods [37]. In Guelph, a university town in Canada, stray cat abundance was highest in residential areas and lowest in commercial and institutional areas, and negatively related to building density [38]. Reported cat population sizes (relative to human population size) vary widely, but are typically in the order of 78 to 168 cats/1000 human residents in urban environments [8,11]. In areas contributing disproportionately to shelter intake and euthanasia, a phone survey estimated there were 236 fed community cats/1000 human residents and the cat density was 21 cats/km^2^ [9], whereas cat densities are lower in forested areas distant from human habitation (0.034 cats/km^2^) [6]. A starting estimate of 67 free roaming cats fed on a daily basis per 1000 residents is recommended, and adjusted up or down based on climate, housing density (cat numbers lower if high density apartment housing), and local knowledge [10,39]. It is reasonable to conclude that more cats are found in urban areas where there is a high degree of human interaction. 

### 4.2. Colony Characteristics and Change over Time

Our median initial colony size of 11.5 cats for 44 colonies was similar to the mean cat colony sizes reported from North Carolina (13 cats) [14] and Florida (7 cats) [30]. In our study, with individual-operated programs, the median percent decline in total number of cats was 40% over a median of four years, most of which was attributable to adult cats and kittens being removed for adoption, combined with desexing to prevent rebound of the population. With individual-operated programs, the median number of cats removed as a ratio of initial colony size was 0.8; this is equivalent to 80% of the original colony size being removed for adoption, and is higher than in a Florida study where 47% of 155 community cats were adopted from 1991 to 1995 and the majority of the remaining cats were desexed, leading to colony attrition over time [29]. 

For colonies under individual- or organization-operated programs pooled, the median TNR duration of 2.2 years was possibly too short for death and emigration to significantly reduce colony size. These led to substantial attrition in other studies [29]. Rehoming is particularly important when programs are in the initial stages, before age-related attrition occurs, as was the case in the 33% of the colonies in our study managed with TNR for less than one year. In our study, approximately one third of colonies did not decrease in size, and this was mainly attributable to fewer cats and kittens being removed for adoption (<1% versus 56% in colonies that did decrease), and shorter time for a decrease through attrition to occur (0.5 years versus 3.2 years) because desexing rates were only slightly lower (65% versus 75%). Nearly all respondents in our study removed kittens up to 12 weeks of age for adoption or socialization before adoption, and 83% removed adoptable adult cats, with a median of 3.5 cats and 8 kittens removed per colony in the previous 12 months. In a TNR program involving 132 colonies in Florida that occurred over nine months, 26% of cats were adopted from the colonies representing a mean of 1.9 cats per colony [30]. This was the biggest cause of colony attrition, followed by death (16%) and disappearance (16%). As in our study, removal of adult cats and kittens for adoption is crucial if early reduction in colony size is to be achieved [9].

We did not collect data on immigration of cats into the colonies. However, immigration is a major factor contributing to colony size [40]. In an Israeli study over one year that monitored desexed and entire colonies that were fed, despite initially desexing all cats in the desexed colonies, colony size increased compared to the entire cat colonies, because sexually intact cats more readily immigrated into desexed colonies, and desexed cats were less likely to emigrate [40]. Desexing immigrant cats is important to control colony size in TNR programs.

Most respondents described attempting to desex the entire population over a short period and at the time of reporting, the median percent of all cats that were desexed was 100% in colonies with individual-operated programs. Based on medians, despite rehoming over four years the equivalent of 80% of the original colony size with individual-operated programs, the decrease in colony size by 37% suggests that there was some inflow from births and net immigration. In a Florida study, only 56% of cats were desexed over nine months of the program [30]. Colony size experienced immigration and births from entire cats, but the mean colony size was 27% lower after four years. 

Matrix population models have indicated that population size does not increase when between 51% and 94% of all cats are desexed [9] and least 75% to 80% of all cats must be desexed to cause population decline and eventual colony extinction, assuming that immigrant cats are also desexed [15]. The mean calculated time to extinction of 12.8 years fitted with the observed decline in three colonies over seven years [15], and also with the observed decline in one colony in our study on the University of New South Wales campus, where a reduction of 71% (77 to 22 cats) occurred over eight years. In this colony, adoptable cats were initially removed and immigrants identified with motion-detecting cameras, trapped and desexed. Other studies report colony attrition, for example on the University of Florida campus, a colony decreased by 66% (68 to 23 cats) over 11 years and no kittens were observed after the first four years of operation [29].

Based on the results of our study and studies from the USA, desexing the entire colony over a short period may not occur [30], and percentages of cats that are desexed may not be sufficiently high for population attrition [15]. Because community sources of funding largely pay for desexing and care of the colonies, and respondents reported that lack of resources was a challenge, it would behoove welfare agencies and municipalities to provide support to community groups to ensure the majority of colonies are desexed over a short period, to facilitate colony attrition and reduce shelter intake of stray cats [9]. 

In our study, desexing occurred at private veterinary clinics. This contrasts with large-scale, non-profit TNR programs in the USA which utilize clinics that desex more than 100 cats per day, and operate on an intermittent basis utilizing volunteers, including veterinary students [41]. The large scale of these operations decreases surgical costs per cat [41], and their desexing is augmented by people taking cats to private veterinary clinics for desexing as part of ongoing daily operations, as occurred in our study [41].

### 4.3. Reasons Respondents Became Involved in TNR

Overwhelmingly, respondents were motivated to engage in TNR to stop breeding (77% of respondents) and because of welfare reasons, for example to make life better for the cats (67%). This is consistent with the purpose of TNR programs, which is to reduce the numbers of cats and improve their health [42]. TNR can improve cat welfare as shown by improved body condition of cats in TNR programs, and body condition scores can be comparable to owned cats [43]. In that study of 105 adult cats in a TNR program in Florida, body weight increased by 40% and body condition score increased by 1 unit (on a scale of 1 to 9) one year after desexing [43]. TNR programs are also associated with increased longevity of cats, with 83% remaining on site after six years compared to 42% of pet cats still in their home after five years [44]. Paradoxically, TNR may be considered as abandoning cats, and therefore illegal under animal welfare legislation in Australia. This is not supported by our study, where cats were fed once or twice a day, and were often vaccinated and given other preventative health care.

Although we did not explore other personal reasons for TNR, in an Australian internet survey, of respondents feeding stray cats, most somewhat or strongly agreed with the statements that feeding a stray cat makes me feel good (87%), people who are important to me would approve of me feeding a stray cat (76%), and feeding a stray cat is the right thing to do (58%) [20]. 

We also did not specifically ask if motivation was to decrease euthanasia of cats in shelters and municipal pounds, but 60% of respondents said it was because the cats were at risk of being trapped and killed by authorities. Stray cats admitted to shelters often have a poor outcome, although only 10% of stray cats admitted to the largest welfare agency in Australia, the Royal Society for Prevention of Cruelty (RSPCA), were classed as feral based on behavior [23,24]. This indicates that most cats euthanized in Australian shelters and municipal pounds are not feral, and is consistent with responses in the Australian internet survey, where most respondents who fed stray cats perceived the cats as friendly (63%) or neither friendly or unfriendly (29%) [20]. TNR targeted to locations of high shelter intake has been shown to reduce shelter intake and numbers of cats euthanized [8]. In Florida, a TNR-targeted area where 60 cats/1000 human residents were desexed over two years (54% of the unowned cat population) was compared with outcomes in the control area where 8 cats/1000 human residents were desexed. Based on data collected at the end of the second year, in the targeted area, shelter intake of cats decreased by 66% from baseline figures, compared to a 12% decrease in the control area, and intake was 3.5 times higher, and the number euthanized was 17.5 times higher in the control area compared to the targeted area [9]. Dog intake in the target area also decreased compared to the control area (36% decrease compared to 9%).

### 4.4. Feeding and Shelter Provision

At least two-thirds of respondents fed cats at a colony with which they were involved and the most time-consuming activity reported by respondents in both individual- and organization-operated programs was feeding cats. Cats were fed at least once and often twice daily. Regular feeding improves cat welfare and decreases wildlife predation [45]. However, the majority of respondents did not remove uneaten food at the end of feeding time. Leaving uneaten food encourages scavengers including rats to aggregate [46], increasing the risk of nuisance complaints, and may increase the risk of transmitting zoonotic diseases [47]. Removal of food after 30 to 60 min minimizes the risk of attracting other animals, and together with a consistent feeding time is recommended by the ASPCA and other organizations providing guidelines for management of community cats [48]. A short and consistent feeding time also facilitates monitoring health of cats at the feeding station, and identification of immigrant cats.

Other recommendations for feeding include the provision of a feeding station that protects cats from the weather, and is situated away from areas of high human traffic to minimize complaints [46]. Respondents provided shelter more often in individual-operated programs compared with organization-operated programs, and this usually consisted of cardboard boxes or modified canine kennels. In Florida, shelter was provided by 75% of caretakers and comprised mainly of existing housing such as the porch (40%) or shed (32%) [30].

### 4.5. Trapping Effort

In our study, conditioning cats to a feeding schedule reduced the typical number of nights required to trap a cat, with 80% of respondents reporting it took two or fewer nights. This is in contrast to a study of nine colonies in rural (farm) locations involving between 10 and 27 cats each, where feeding cats for three days in the traps did not subsequently reduce trapping success in the first five nights trapping in the colony. It took a mean of six trap-nights to trap a cat, and an average of 8.9 trap-nights to trap each cat until 90% of the colony were captured [15]. In contrast, trapping feral cats at two open forest sites yielded up to 2.6 cats per 100 trap-nights, or at least 38 nights per trap to catch one cat [6]. In our study, respondents were asked to report their trapping activities as at the time they completed the questionnaire. Therefore, we did not account for the maturity of the TNR colony and responses reflected a broad range of colonies from old to new, and hence differing lengths of interaction with the cats. Furthermore, our findings were derived from respondents’ recollections of trapping time inputs, instead of prospective measurement. Nevertheless, most respondents found conditioning with food effective in reducing trapping effort, which is important because 91% of respondents reported sitting nearby, waiting until the cat was trapped. 

### 4.6. Healthcare

Although in some jurisdictions in Australia, TNR is considered neglect and illegal under legislation related to animal welfare, the care provided to cats in the colonies reported in our study is not consistent with abandonment of cats. Many respondents provided treatment for fleas and intestinal parasites for adults and kittens, and vaccinated most kittens for herpes virus, calicivirus and panleukopenia virus. Testing and vaccination for FIV and FeLV however, were less commonly provided. The prevalences in Australia of FIV and FeLV in relinquished shelter cats are 6% and 1%, respectively, but these are higher in sick cats and vary by location [49,50]. In two colonies of cats in periurban (*n* = 48) and inner city Sydney (*n* = 20), apparent prevalences of FIV (i.e., proportions testing positive) were 21% and 25%, respectively [50]. In situations where the prevalence is low, a high proportion of FIV and FeLV test positives are false positive results (i.e.; the positive predictive value is low) and so ELISA test-positive cats should then undergo confirmatory testing such as Western blot [49,50,51] or PCR [52]. Recommendations for cats in TNR programs vary from not routinely testing healthy cats due to cost and false positives and only testing sick cats with signs consistent with FIV or FeLV [53], to testing and either euthanizing or rehoming to single cat households [51,53]. Current recommendations by the American Association of Feline Practitioners are to not test cats in TNR programs and to use resources for higher priorities such as desexing (Levy et al., 2008). Wound and abscess treatment were the most common additional treatments provided when necessary. Catfight abscesses or skin wounds were also the most common health care issues requiring treatment in a US study [15]. 

The proportion of cats receiving healthcare in our study exceeded that reported in Florida, where only 26% of caretakers reported providing basic vaccinations and deworming for cats, and even less for other treatments such as antibiotics, ear medication and flea treatments [30]. In other reported programs, vaccination and anthelmintic treatments were used [54,55]. 

In our study, 44% of respondents microchipped the cats when they were desexed and recorded their name on the database. In an Australian study of cat semi-owners, some (20%) also reported microchipping cats, which is traditionally considered indicating ownership, although these respondents also did not perceive themselves as owners of the cats [20]. In our study, only one third of respondents ear-tipped cats, a recommended procedure in North America. Ear tipping is used on free-living cats to indicate that they have been desexed, and helps to protect cats and minimize unnecessary retrapping [46,56]. The low ear tipping rate in our study might reflect state and federal legislation in Australia relating to invasive species which prohibits the release of feral cats. It is possible respondents in our study were unwilling to alert authorities to their activities by using ear tipping. 

### 4.7. Funding

Most respondents involved in individual-operated programs contributed by either cash and/or in-kind for transport, desexing, traps, bedding and food. However, private sources (other than the respondent) and/or organizations also assisted with cash and/or in-kind support for desexing, and traps and food in individual-operated programs. The proportion of respondents that self-funded may be high because large organizations such as the RSPCA and Australian Veterinary Association have official policies against TNR [56,57], and it is considered illegal in most Australian States. This is in contrast to USA, where TNR is funded by philanthropic organizations [29], many welfare agencies and municipal governments. However, some municipalities in USA have laws which effectively make TNR illegal, for example, with feeding bans and confinement laws (per com Bryan Kortis, Neighborhood Cats). Legalizing high quality TNR would facilitate funding and in-kind support from philanthropic, animal welfare and government sources. 

### 4.8. Limitations

One of the major limitations of our study was that 33% of the TNR colonies were managed for less than one year, with 10 of those 13 colonies being under an organization-managed program. Hence, our data from organization-managed programs were not suitable for assessing the long-term outcomes of TNR. 

Furthermore, our approach to enrolling participants via convenience and snowball sampling was prone to selection bias, reducing confidence about inferences about the entire population of people involved in TNR activities across Australia. Our enrolled participants were likely highly motivated group of caretakers which might have influenced the data they shared. Participants were encouraged to describe all colonies they were involved in, although they were not obligated to. As a result, respondents may have reported on the colonies that demonstrated the most success. About half of our respondents were from Sydney. Although Sydney is the most populous city in Australia, over-representation of people involved in TNR from Sydney seems likely. This was probably because Maggie’s Rescue (our primary source of respondents) is based in Sydney, New South Wales.

### 4.9. Recommendations

Currently in some jurisdictions, it is illegal without a permit to adopt, feed or return urban stray kittens or cats because they are defined as feral [58,59]. We recommend that legislation relating to invasive pest species be amended to define feral cats subject to the legislation as those having no reliance on humans for food or shelter, and to specifically exclude those cats that have some reliance on humans for food or shelter (urban strays). Legislative change is essential to facilitate effective management practices to reduce numbers of urban stray cats, cat-related complaints, predation of wildlife, and cat intake and euthanasia in municipal pounds and shelters and its associated costs and impact on staff. 

Based on the results of our study and international literature on TNR, we recommend that TNR be legalized for urban stray cats in Australia, and encouraged by government authorities and welfare agencies to reduce cat numbers. A registry of TNR colonies should be developed at the state level by the major welfare agency in Australia, the RSPCA, and participation encouraged through access to low-cost desexing, microchipping, cat traps, food and funding. The register should include the colony carers’ primary and secondary contact details, any affiliated organization, the location of colony including address and type of site, and an annual statement of cat numbers including desexed/entire, and cats/kittens. This would facilitate assessing the efficacy of TNR in controlling population sizes, and identifying where more resources need to be deployed. 

We recommend TNR colonies are managed with an identified carer rather than unmanaged TNR colonies, so ongoing care can be provided. However, if cats are being adequately fed based on body condition, but no specific individual or organization can be identified as a carer, it is recommended desexing of cats proceed. The health of cats in unmanaged TNR colonies is also acceptable as only 4% were emaciated compared with 2% of pet cats, and cats in managed colonies [60]. We recommend that initially all adoptable kittens and cats be removed and adopted, when available foster and adoption resources allow, obtaining a rapid reduction in colony size. As close as possible to 100% of the remaining healthy cats should be desexed within six months (one week ideally) and returned to the colony location. Immigrant cats should be desexed soon after arrival. For best practice, cats should also be vaccinated, and provided with parasite control at desexing, and minor health treatments provided if indicated. Cats should be microchipped, and details of the carers’ primary and secondary contacts plus any associated organization registered on the microchip database. Ear tipping is recommended to protect cats by facilitating rapid identification of cats being managed in TNR programs. Food should be placed in containers hidden from public view, with uneaten food removed after 30–60 min. If shelter is provided, it should be appropriate for the climate and hidden from public view. It is recommended that educational material be developed by municipal councils and/or welfare organizations, and distributed to people in colony areas to minimize complaints. Best practice should be implemented on dispute resolution, including offering an opportunity for temporary rental of cat deterrent devices to individuals concerned about cats or colonies of cats on their properties.

## 5. Conclusions

TNR activities are occurring in all major mainland capital cities of Australia, with most respondents involved with colonies in New South Wales and Victoria, and operating as individuals rather than associated with organizations. Colony sizes decreased a median of 31% over two years from 11.5 to 6.5 cats. Adoption of kittens and adoptable adults was a key factor in an early reduction of colony size. Despite the intention to desex 80% to 100% of the colony, on average only 50% of respondents achieved this within the first six months. In addition to trapping, neutering and returning desexed cats to the colonies, many respondents committed substantial time to caring for the colonies, with feeding and locating resources consuming the most time. Cat and kitten health was likely improved as desexing, prophylactic health care, and other health care were commonly provided. Therefore, based on our results, TNR is a useful method of non-lethal control of urban stray cat populations, and generally results in reduced colony size and improved health care. It is primarily funded by the individuals involved and other private sources. Therefore, it has the opportunity to be more cost-effective for municipalities than the current practice of trapping and killing. We recommend TNR be legalized in Australia for urban stray cats, and guidelines developed for best practice, including desexing as close to 100% of the colony as possible, and adoption of kittens and friendly adults to achieve rapid reduction in cat numbers. Greatest impact occurs when TNR is targeted to locations over-represented by cat intake into shelters and municipal pounds, and by cat-related complaints, and we recommend that funding for TNR be prioritized to these areas.

## Figures and Tables

**Table 1:** Australian states where respondents were involved with managing colonies by trap, neuter and return (TNR) (*n* = 51).

State/Territory	Respondents in Individual-Operated Programs (*n* = 29)	Respondents in Organization-Operated Programs (*n* = 21)
New South Wales	15 (56%)	11 (52%) ^2^
Victoria	6 (22%)	5 (24%)
Western Australia	3 (11%)	1 (5%)
Queensland	2 (7%)	2 (10%)
Australian Capital Territory	1 (4%)	0 (0%)
South Australia	0 (0%)	2 (10%)
State not specified	2 ^1^	

^1^ A further two respondents did not report whether they were involved in TNR as individuals or organizations; one was in Western Australia and no state was specified by the other; ^2^ One of these respondents was also involved with one or more colonies in Australian Capital Territory.

**Table 2:** Location of colonies (*n* = 98).

Locations	All Colonies (*n* = 98) ^1^	Colonies in Individual-Operated Programs (*n* = 49)	Colonies in Organization-Operated Programs (*n* = 46)
Private residential homes	25 (26%)	14 (29%)	10 (22%)
Industrial areas or factory complexes	20 (20%)	9 (18%)	11 (24%)
Alleyways or streets	13 (13%)	6 (12%)	6 (13%)
Government housing complexes	8 (8%)	4 (8%)	4 (9%)
Vacant blocks or vacant buildings	7 (7%)	3 (6%)	3 (7%)
Parks and reserves	5 (5%)	2 (4%)	3 (7%)
Universities	5 (5%)	1 (2%)	4 (9%)
Food outlet or shopping	4 (4%)	1 (2%)	3 (7%)
Private housing complexes	3 (3%)	3 (6%)	0 (0%)
Schools	2 (2%)	2 (4%)	0 (0%)
Other	6 (6%)	4 (8%) ^2^	2 (4%) ^3^

^1^ One further respondent reported three colony locations but did not report whether they were involved in TNR as an individual or organization. ^2^ These locations were a train station, a long-stay caravan park, a council car park and a mixed area. ^3^ These locations were a community facility and an assisted-care housing complex.

**Table 3:** Colony size and composition prior to TNR being commenced and at the time the respondent completed the questionnaire (“current”) for 21 colonies managed using TNR where unambiguous numbers were reported (median (range)).

Colony Size & Composition	All Colonies (*n* = 21)	Colonies Under Individual-Operated Programs (*n* = 8)	Colonies Under Organization-Operated Programs (*n* = 13)	*p* ^1^
Number of cats in colony at commencement of TNR	11 (3 to 40)	12.5 (4 to 40)	10 (3 to 20)	0.478
Number of cats & kittens removed for adoption, since TNR began in colony:				
Number	3 (0 to >50)	7 (3 to >50)	1 (0 to 19)	0.013
Number as ratio of number of cats in colony at commencement of TNR	0.3 (0.0 to 1.9)	0.8 (0.2 to >1.2)	0.1 (0.0 to 1.9)	<0.001
Current situation				
Total number of cats	5 (1 to 23)	6.5 (1 to 23)	5 (3 to 15)	0.896
Percent desexed	69% (21 to 100)	100% (21 to 100)	67% (25 to 100)	0.192
Percent adult (older than six months)	100% (47 to 100)	100% (65 to 100)	100% (47 to 100)	0.928
Percent male ^2^	50% (0 to 100)	38% (0 to 67)	60% (0 to 100)	0.028
Time TNR had been used in the colony (years)	1.7 (0.1 to 12)	3.2 (0.4 to 12)	1.0 (0.1 to 11)	
Percent reduction in number of cats in colony	40% (−77% to 83%)^3^	55% (−77% to 83%)	24% (−67% to 70%)	

^1^ P for comparison of distributions between colonies under individual-operated and organization-operated programs; ^2^ Expressed as a percentage of those whose sex was known; ^3^ “−77%” equates to a 77% increase in the number of cats in the colony.

**Table 4:** Reasons respondents gave for becoming involved in TNR by 30 respondents that selected at least one option. Respondents could choose multiple reasons and 23 (77%) selected three or more options.

Reason for Becoming Involved with TNR	All Respondents (*n* = 30 ^1^)	Individual-Operated Program (*n* = 17)	Organization-Operated Program (*n* = 11)
I wanted to stop the breeding	23 (77%)	13 (76%)	10 (91%)
This makes life better for stray cats	20 (67%)	11 (65%)	9 (82%)
The cats were at risk of being trapped and killed by the authorities	18 (60%)	10 (59%)	8 (73%)
I have cared for stray cats for a long time, and this is the best strategy to manage them	15 (50%)	8 (47%)	7 (64%)
I live nearby and I wanted to help the cats	14 (47%)	9 (53%)	5 (45%)
I could find no-one else to help the cats	11 (37%)	9 (53%)	2 (18%)
I wanted to help the sick cats and kittens	11 (37%)	7 (41%)	4 (36%)
TNR is used by the animal welfare organization I belong to	8 (27%)	2 (12%)	5 (45%)
I was asked to help by a friend or acquaintance	7 (23%)	4 (24%)	3 (27%)
The stray cats were a nuisance	6 (20%)	3 (18%)	2 (18%)

^1^ Two respondents did not report whether they were involved in TNR as individuals or organizations.

**Table 5:** Time commitments (median hours (range)) by respondents in various TNR activities, and time commitments and numbers of people providing care for the largest colony **^1^** for each role; only time commitments >0 and numbers >0 were included in analyses

Role Undertaken at the Colony	Hours that Respondent Cared for Colonies			Hours that Respondent and Others Cared for Largest Colony ^1^			Number of Carers Caring for Largest Colony ^1^		
	All Respondents	Individual-Operated Program	Organization-Operated Program	All Respondents	Individual-Operated Program	Organization-Operated Program	All Respondents	Individual-Operated Program	Organization-Operated Program
Feeding cats (h/week)	4 (0.5 to 20) *n* = 20	3 (1 to 6) *n* = 4	4 (0.5 to 20) *n* = 15	6.5 (1 to 25) *n* = 14	8.5 (5 to 25) *n* = 6	3.5 (1 to 2) *n* = 7	3 (1 to 12) *n* = 13	3 (1 to 12) *n* = 7	2 (1 to 7) *n* = 6
Organizing feeding roster (h/week)	1.5 (0.1 to 5) *n* = 8	2.5 (0.5 to 3) *n* = 4	0.7 (0.1 to 5) *n* = 4	1 (0.4 to 10) *n* = 8	1 (0.5 to 2) *n* = 5	6 (0.4 to 10) *n* = 3	1 (1 to 5) *n* = 8	1 (1 to 3) *n* = 6	3 (1 to 5) *n* = 2
Locating resources (h/month)	4.5 (0.5 to 160) *n* = 22	4 (2 to 24) *n* = 7	5 (0.5 to 160) *n* = 14	7 (1 to 20) *n* = 13	8.5 (2 to 15) *n* = 6	6 (1 to 20) *n* = 6	2.5 (1 to 6) *n* = 10	2.5 (2 to 6) *n* = 6	3 (1 to 6) *n* = 4
Developing/maintaining community relations (h/month)	2 (1 to 18) *n* = 17	2 (1 to 16) *n* = 7	2 (1 to 18) *n* = 10	6 (1 to 20) *n* = 11	6.5 (1 to 20) *n* = 6	2 (1 to 20) *n* = 5	2 (1 to 4) *n* = 9	2.5 (1 to 4) *n* = 6	1 (1 to 4) *n* = 3
Finding homes for cats and/or kittens (h/6 months)	20 (2 to 100) *n* = 16	25 (2 to 100) *n* = 8	11 (3 to 60) *n* = 8	17.5 (2 to 200) *n* = 10	40 (10 to 200) *n* = 6	8.5 (2 to 50) *n* = 4	3 (1 to 6) *n* = 10	3 (2 to 6) *n* = 7	2 (1 to 5) *n* = 3
Trapping (h/6 months)	1 (1 to 100) *n* = 16	21 (6 to 100) *n* = 8	10 (1 to 20) *n* = 8	26 (10 to 100) *n* = 9	38 (20 to 100) *n* = 6	10 (10 to 50) *n* = 3	4 (1 to 6) *n* = 10	4 (2 to 6) *n* = 7	1 (1 to 4) *n* = 3
Returning to location (h/6 months)	8 (1 to 100) *n* = 14	4.5 (2 to 50) *n* = 8	10 (1 to 100) *n* = 6	10 (5 to 100) *n* = 10	10 (5 to 30) *n* = 6	30 (5 to 100) *n* = 4	3 (1 to 100) *n* = 10	3 (2 to 4) *n* = 6	2.5 (1 to 10) *n* = 4
Transporting to vet for desexing and back to colony (h/6 months)	8 (2 to 40) *n* = 18	10 (2 to 40) *n* = 9	6 (2 to 10) *n* = 9	20 (7 to 60) *n* = 9	20 (10 to 60) *n* = 6	10 (7 to 50) *n* = 3	2 (1 to 4) *n* = 9	3 (2 to 4) *n* = 6	1 (1 to 2) *n* = 3
Transporting cats/kittens for rehoming (h/6 months)	10 (1 to 30) *n* = 17	10 (2 to 30) *n* = 8	10 (1 to 25) *n* = 9	15 (1.5 to 60) *n* = 8	20 (10 to 60) *n* = 5	10 (1.5 to 50) *n* = 3	2 (1 to 5) *n* = 9	3 (2 to 5) *n* = 6	1 (1 to 2) *n* = 3

**^1^** The largest colony that the respondent was involved with.

**Table 6:** Provision of food and shelter at a TNR colony the respondent was involved for 25 respondents that fed cats at a TNR colony. Percentages are for the 25 respondents who indicated they fed cats at a TNR colony they reported on. ^1^

	All Respondents that Fed (*n* = 25) ^2^	Individual-Operated Program (*n* = 15)	Organization-Operated Program (*n* = 8, *Feeding Frequency n* = 9)
*Feeding frequency*			
Once daily	17 (68%)^1^	8 (53%)	8 (89%)
Twice daily	8 (32%)	7 (47%)	1 (11%)
*Feeding times*			
During daylight hours	12 (50%)	8 (53%)	3 (38%)
After dark or early morning	8 (33%)	5 (33%)	3 (38%)
Various times of the day	4 (17%)	2 (13%)	2 (25%)
*Use of specialized feeding stations to hide food*			
Yes	15 (63%)	10 (67%)	5 (63%)
No	9 (38%)	5 (33%)	3 (38%)
*Food placement*			
In a container above ground level	3 (13%)	2 (13%)	(0%)
In a container on the ground	11 (46%)	6 (40%)	5 (63%)
Both	2 (13%)	1 (9%)	1 (33%)
On the ground	8 (33%)	6 (40%)	2 (25%)
*Food left to be eaten at any time*			
Yes	16 (67%)	9 (60%)	7 (88%)
No	8 (33%)	6 (40%)	1 (13%)
*Provision of shelter*			
Yes	12 (50%)	10 (67%)	2 (25%)
No	12 (50%)	5 (33%)	6 (75%)

^1^ The minimum percentages for all 39 respondents can be calculated by multiplying percentages in Table 4 by 25/39 (i.e., by about two-thirds); these would be correct if none of the other 14 respondents provided food or shelter. It was unknown whether any of the remaining 14 respondents fed cats. ^2.^ One respondent answered at least one question about feeding and shelter at a TNR colony but did not specify whether they were involved in an individual- or organization-operated program.

**Table 7:** Routine health care provided to adult cats (those aged above six months) and kittens for 26 respondents that provided health care at a TNR colony they was involved with.

Health Care	Adult Cats (% of 26 Respondents) ^1^	Kittens (% of 26 Respondents) ^1^
Treatment for fleas	18 (69%)	20 (77%)
Treatment for intestinal parasites	19 (73%)	20 (77%)
F3 and/or F4 vaccination ^2^	10 (38%)	16 (62%)
Test for FIV (Feline Immunodeficiency Virus)	5 (19%)	9 (35%)
Vaccination for FIV	5 (19%)	5 (19%)
Test for FeLV (Feline Leukemia virus)	2 (8%)	7 (27%)
Vaccination for FeLV	2 (8%)	2 (8%)

^1^ The minimum percentages for all 39 respondents can be calculated by multiplying percentages in Table 7 by 26/39 (i.e., by about two-thirds). These would be correct if none of the other 13 respondents provided health care. ^2^ F3 vaccination consists of feline herpes virus, calicivirus and panleukopenia virus; F4 vaccination consists of F3 and chlamydia.

**Table 8:** Proportions of respondents involved with individual- and organization-operated programs whose activities were self-funded, privately funded or organization funded with cash and in-kind donations; each respondent could select multiple funding sources.

Activity	Individual-Operated Program									Organization-Operated Program								
	No. Respondents ^1^	Self-Funded—Cash	Self-Funded—in Kind	Self-Funded Pooled	Privately Funded by Others—Cash	Privately Funded by Others—in Kind	Organization Funded—Cash	Organization Funded—in Kind	Privately or Organization Funded Pooled ^2^	No. Respondents ^1^	Self-Funded—Cash	Self-Funded—in Kind	Self-Funded Pooled	Privately Funded by Others—Cash	Privately Fundedby Others—in Kind	Organization Funded—Cash	Organization—in Kind	Privately or Organization Funded Pooled ^2^
Transport	16	16 (100%)	2 (13%)	16 (100%)	1 (6%)	0 (0%)	1 (6%)	1 (6%)	2 (13%)	9	4 (44%)	3 (33%)	6 (67%)	1 (11%)	2 (22%)	1 (11%)	0 (0%)	4 (44%)
Desexing	16	15 (94%)	1 (6%)	15 (94%)	2 (13%)	0 (0%)	5 (31%)	1 (6%)	7 (44%)	10	3 (30%)	1 (10%)	3 (30%)	3 (30%)	2 (20%)	8 (80%)	1 (10%)	10 (100%)
Traps	16	14 (88%)	0 (0%)	14 (88%)	0 (0%)	0 (0%)	2 (13%)	2 (13%)	4 (25%)	10	4 (40%)	1 (10%)	4 (40%)	3 (30%)	4 (40%)	5 (50%)	0 (0%)	9 (90%)
Bedding	16	13 (81%)	1 (6%)	13 (81%)	2 (13%)	0 (0%)	1 (6%)	0 (0%)	3 (19%)	7	4 (57%)	3 (43%)	6 (86%)	4 (57%)	3 (43%)	3 (43%)	1 (14%)	7 (100%)
Food	16	16 (100%)	2 (13%)	16 (100%)	2 (13%)	1 (6%)	1 (6%)	1 (6%)	4 (25%)	10	6 (60%)	2 (20%)	6 (60%)	5 (50%)	4 (40%)	3 (30%)	2 (20%)	9 (90%)

^1^ No. respondents that selected at least one funding source from within each activity; 2 additional respondents did not specify whether they were involved in an individual- or organization-operated program; these respondents were not included in this table; ^2^ Either cash and/or in kind.
